# Thermal and flow performance analysis of a concentrated linear Fresnel solar collector with transverse ribs

**DOI:** 10.3389/fchem.2022.1074581

**Published:** 2023-01-04

**Authors:** Husam Abdulrasool Hasan, Jenan S. Sherza, Azher M. Abed, Hussein Togun, Nidhal Ben Khedher, Kamaruzzaman Sopian, Jasim M. Mahdi, Pouyan Talebizadehsardari

**Affiliations:** ^1^ Department of Air Conditioning and Refrigeration Techniques, AL-Esraa University College, Baghdad, Iraq; ^2^ Air conditioning and Refrigeration Techniques Engineering Department, Al-Mustaqbal University College, Babylon, Iraq; ^3^ Department of Biomedical Engineering, College of Engineering, University of Thi-Qar, Thi-Qar, Iraq; ^4^ College of Engineering, University of Warith Al-Anbiyaa, Karbala, Iraq; ^5^ Department of Mechanical Engineering, College of Engineering, University of Ha’il, Ha’il, Saudi Arabia; ^6^ Laboratory of Thermal and Energetic Systems Studies (LESTE) at the National School of Engineering of Monastir, University of Monastir, Monastir, Tunisia; ^7^ Solar Energy Research Institute, University Kebangsaan Malaysia, Selangor, Malaysia; ^8^ Department of Energy Engineering, University of Baghdad, Baghdad, Iraq; ^9^ Centre for Sustainable Energy Use in Food Chains, Institute of Energy Futures, Brunel University London, Uxbridge, Middlesex, United Kingdom

**Keywords:** thermal enhancement, compound parabolic collector, ribs, solar thermal system, Nusselt number, friction coefficient

## Abstract

This article deals with the impact of including transverse ribs within the absorber tube of the concentrated linear Fresnel collector (CLFRC) system with a secondary compound parabolic collector (CPC) on thermal and flow performance coefficients. The enhancement rates of heat transfer due to varying governing parameters were compared and analyzed parametrically at Reynolds numbers in the range 5,000–13,000, employing water as the heat transfer fluid. Simulations were performed to solve the governing equations using the finite volume method (FVM) under various boundary conditions. For all Reynolds numbers, the average Nusselt number in the circular tube in the CLFRC system with ribs was found to be larger than that of the plain absorber tube. Also, the inclusion of transverse ribs inside the absorber tube increases the average Nusselt number by approximately 115% at Re = 5,000 and 175% at Re = 13,000. For all Reynolds numbers, the skin friction coefficient of the circular tube with ribs in the CLFRC system is larger than that of the plain absorber tube. The coefficient of surface friction reduces as the Reynolds number increases. The performance assessment criterion was found to vary between 1.8 and 1.9 as the Reynolds number increases.

## Introduction

Improving the thermal efficiency of solar thermal systems by enhancing the heat transfer rates between the working fluid and the carrying tube has been the focus of several research investigations over the last few decades. Many technical processes were applied to develop the total efficiency of different solar water-heating systems. The passive technique is unique among all the heat transfer improvement approaches. This technique entails increasing the turbulent motion in the solar thermal collector to increase the overall efficiency of the system. Recently, many heat transfer improvement approaches have been established and extensively used across a range of technical applications and industrial systems, including parabolic-trough solar thermal collectors, evacuated tube systems, flat-plate solar thermal systems, domestic solar hot water systems, double-pipe heat exchangers, power plants, cooling systems, heating systems, solar thermal collectors, and solar water heaters (Hasan et al., 2018a; Sopian et al., 2018). Mohammed et al. (2013a) numerically examined the impact of louvered strips on the thermal performance and heat transfer enrichment in a double-pipe heat exchanger employing changed types of nanofluids as working fluids. Many arrangements of louvered strips were tested, and it was found that the forward louvered strips provided approximately 367%–411% higher heat transfer enhancement when the incline angle was 30°. Mohammed et al. (2013b) numerically studied a transversely corrugated tube with many geometrical parameters and its performance evaluation criterion, heat transfer, and fluid flow under different Re ranging from 5,000 to 60,000. Different designs of transversely corrugated tubes were calculated with different relative roughness heights between e/d = 0.025 and e/d = 0.1 and different pitches ranging from p/d = 0.5 to p/d = 1.5, with many relative roughness widths between w/d = 0.05 and w/d = 0.2. The Nusselt number increased with an increase in Reynolds number, the height of roughness, and width, while it decreased with the pitch roughness. The maximum Nu was found to be e/d = 0.1. Using computational fluid dynamics (CFD), Kaood and Hassan (2020) studied the influence of triangular, curved, trapezoidal, and rectangular ribs in corrugated tubes on the thermo-hydraulic performance with different nanofluids. A highest total performance of 37% was found for the curvedly ribbed tube at Re = 10,000 and using flow (GNP-SDBS/DW) nanofluids with a high flow rate. Hasani et al. (2022) numerically investigated the impact of different geometries of grooved channels on the thermo-hydraulic performance of a helical microchannel heat sink. Staggered corrugated tubes in the helical microchannel heat sink provided maximum flow mixing. Comparing the helical microchannel heat sink containing the parallel grooved channel to that containing the smooth channel, the coefficient of heat transfer improved by approximately 70% and 17%, respectively. The parallel grooved channel exhibited the maximum frictional factor. Staggered grooved channels offered maximum performance evaluation criteria (PEC) for the helical microchannel heat sink. The result confirmed that the peak performance evaluation criteria was obtained when the pitch of the groove was 0.75 mm.

Jamshidmofid and Bahiraei (2022) investigated the impact of rectangular ribs in a microchannel heat sink filled with different nanofluids on heat transfer enlargement, and thermal resistance was investigated. The model was run at different Reynolds numbers (100–500) and nanofluid concentrations of 0.05%, 0.1%, and 0.2%. Solid temperature, coefficient of heat transfer, temperature equivalence, and thermal resistance all improved by approximately 14%, 2.35%, 27%, and 25%, respectively. Kumar et al. (2022) numerically examined the influence of wavy heat sink microchannel water—Al_2_O_3_–water nanofluid. Different Reynolds numbers were tested (Re = 100–300). The Al_2_O_3_–water nanofluid presented the highest heat transfer coefficients at maximum concentrations for all values of the Reynolds number. Flow nanofluids at Re = 300 in a wavy heat sink microchannel led to an improvement of the coefficient of heat transfer by approximately 154%. Alqarni et al. (2021) investigated the influence of a novel turbulator (hole–rib) in the tube of parabolic-trough collectors to enhance the overall efficiency of nanofluids with different concentrations of 0, 2, and 4. An approximately 50.5% increase in the thermal efficiency was achieved by increasing the concentration of the nanofluid and the Reynolds number. Alsabery et al. (2021) studied the impact of the flow nanofluid inside a wavy channel for solar heating systems on the thermo-hydraulic performance. The results indicated that the flow of nanofluids had an adequate influence on heat transfer enhancement rates. The average particle velocity and average temperature were found to increase as the channel waviness number increased. Bahiraei et al. (2021a) numerically examined the effect of graphene nanoplatelet nanofluids and ribs on heat transfer in triple-tube heat exchangers. The various geometrical parameters such as rib pitches and heights were considered in this study. The ALO algorithm was the best method for approximating the results. Bahiraei et al. (2021b) examined the effect of crimped/spiral ribs on the thermo-hydraulic performance of an alumina/H_2_O nanofluid flow through a heat exchanger. The highest augmentations achieved for thermal performance and efficiency were approximately 44.91% and 41%, respectively, at a nanofluid concentration of 2%. The heat transfer coefficient was found to increase with increasing pitch and height of ribs in the heat exchanger. The highest thermal performance was obtained at the lowest concentration of nanofluid.

Ben Hamida and Hatami (2021) examined the effect of fins in a channel, considering various hybrid nanofluids, on the thermal-hydraulic performance under an electrical field. Different types of hybrid nanofluids, including CuO–TiO_2_, Al_2_O_3_–TiO_2_, CuO–Al_2_O_3_, and Al_2_O_3_–Cu, were used. Using the nanofluid based on Al_2_O_3_–TiO_2_ led to superior improvement in the average Nusselt number (Nu). It was also noted that an increase in the nanofluid concentration led to a higher Nu, while an increase in the electric voltage led to a higher coefficient of heat transfer. Ekiciler Samet Ali Çetinkaya (2021) studied the effect of different configurations of ribs (triangular, ellipse, and square) in the duct, with different types of nanofluids, on the heat transfer enhancement and fluid flow behavior. The results revealed that the average Nu can be improved by 18.0% using the water–Al_2_O_3_ nanofluid with triangular ribs, and by 32.0% using a hybrid nanofluid (Al_2_O_3_/Cu–water). Elsaid et al. (2021) numerically studied the effect of using different shapes of triple ribs in the tube of the heat exchanger with different types of nanofluids (water–Al_2_O_3_, water–MgO, water–SiC, and water–MWCNT). A hybrid nanofluid of water–Al_2_O_3_+MWCNT provided a higher heat transfer coefficient than that of the single nanofluid. Abu-Hamdeh et al. (2020) improved the thermal efficiency in a cylindrical solar thermal collector by using rectangular ribs in the absorber tube. The numerical results revealed that the rectangular ribs could improve the thermal performance by approximately 47.7% when compared to the plain absorber tube. A relatively large number of studies, including experimental, theoretical, and simulation, have been conducted to reveal the effects of various techniques that can improve the heat transfer enhancement potential in numerous applications such as heat exchangers, solar air heaters, solar thermal systems, solar cooling systems, solar flat-plate collectors, and solar evacuated tube collectors (Al-Shamani et al., 2016; Naje et al., 2016; Al-Waeli et al., 2017; Dezfouli et al., 2017; HameedJaaz et al., 2017; Hasan et al., 2017; Jaaz et al., 2017; Hasan et al., 2018b; Hasan et al., 2018c; Ben Hamida and Hatami, 2021; Rukman et al., 2021; Abdulrasool et al., 2022; Hasan et al., 2022a; Ameen et al., 2022; Hasan et al., 2022b).

The enhancement of heat transfer by using ribs in order to increase the heat transfer coefficient from the flow surface, through an increase in turbulent motion, is the main objective of this article. A novel concept for augmenting the heat transfer rate by using small ribs in a concentrated linear Fresnel solar collector (CLFRC) is tested *via* CFD simulation. Simulation runs were performed to numerically solve the governing thermofluidic equations using the finite volume method (FVM) under different boundary conditions. The key goal is to reveal the impact of using ribs on the heat transfer coefficients and skin friction coefficients in the CLFRC. Transverse ribs are used as they are expected to induce a rapid mixing rate, high turbulent flow, and longitudinal vortex flow. Therefore, enhancement additives in the form of transverse ribs are applied to reduce the size and cost of the concentrated linear Fresnel solar collector, in order to keep up with the rise in fuel prices for power plants as well as the increase in demand for electricity generation. The enhancement also increases the thermal efficiency and hydrodynamic performance of the concentrated linear Fresnel solar collector, which can increase power plants’ total thermal efficiency, reduces carbon dioxide emissions, and mitigates global warming impacts.

## Physical model and mathematical formulation

**FIGURE 1:**
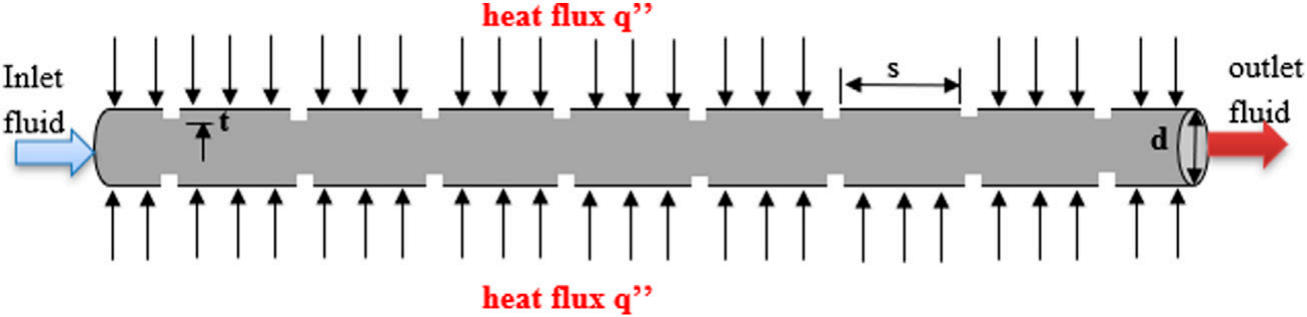
Schematic of the absorber tube with ribs.

**FIGURE 2:**
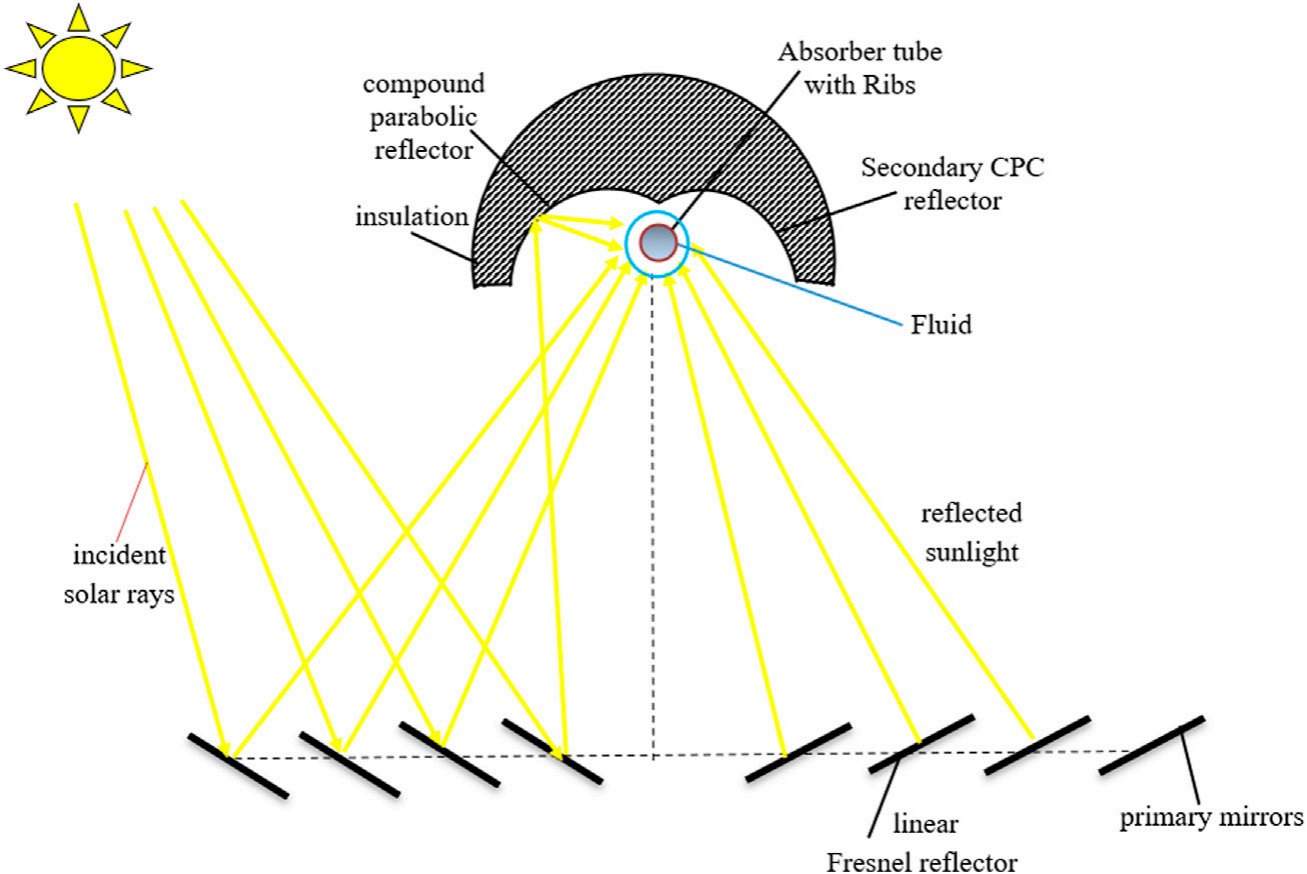
Diagram of the CLFRC system with a secondary CPC.

Figures 1 and 2 show an absorber tube with transverse ribs and a plain absorber tube in a CLFRC. The length of the entrance tube is 100 mm. The effects of the height of the rib $\left(t=0.5\;mm\right)$ and pitch (*p* = 40 mm) are considered for the current analysis. The length of the absorber tube is L = 580 mm, and the diameter is D = 6.6 mm. Water is selected as the working fluid. Figure 1 shows an absorber tube with transverse ribs. Figure 2 presents a linear Fresnel reflector solar thermal system (LFRSTS). The boundary conditions for the absorber tube with transverse ribs in the LFRSTS are detailed for the computational domain, as shown in Figures 1 and 2. These figures also show that a uniform heat flux is applied directly to the upper and lower pipes of the absorber tube with transverse ribs, as well as the plain absorber tube.

The average Nu can be determined (Mohammed, Hasan and Wahid 2013):

$$Nu_{av}=\frac{h_{av}D_{h}}{k_{nf}}.$$ (1)

The hydraulic diameter of the conduit $D_{h}$ and the thermal conductivity of nanofluids $k_{nf}$ can be determined using the following equation:

$$D_{h}=\frac{4A_{C}}{P}.$$ (2)

The cross-sectional area and the flow’s wetted perimeter are denoted by A_c_ and P, respectively.

The friction factor is

$$f=\Delta p\frac{D_{h}^{2}}{L\rho u^{2}}.$$ (3)

Additionally, the Reynolds number can be given as follows:

$$Re=\frac{\rho uD_{h}}{\mu },$$ (4)

where L is the length of the tube, ρ is the density of the nanofluid, u is the velocity of the fluid, and Δp is the differential pressure drop at the computational domain.

The turbulent low channel Dittus–Boelter equation is as follows:

$$Nu_{Dh}=0.023\;Re_{Dh}^{0.8}Pr^{0.4}.$$ (5)

Here, Dh is the hydraulic diameter, Re is the Reynolds number, Pr is the Prandtl number, and Nu is the Nusselt number.

## Numerical procedure

A mathematical model (continuity, momentum, and energy) must be established in order to carry out the numerical simulation. The CLFRC with a secondary compound parabolic collector (CPC) is studied using Ansys Fluent as fluid simulation software to determine the effects of adding transverse ribs to the absorber tube on the thermal and flow performance coefficients in the CPVT collector. The Navier/Stokes expressions in two dimensions with equations of continuity, momentum, and energy determine the phenomena. The Navier/Stokes expressions computationally represent momentum and mass for Newtonian fluids.

$$\frac{\partial }{\partial x_{i}}\left(\rho u_{i}\right)=0,$$ (6)

$$\frac{\partial \left(\rho u_{i}u_{j}\right)}{\partial x_{j}}=-\frac{\partial p}{\partial x_{i}}+\frac{\partial }{\partial x_{j}}\left[\mu \left(\frac{\partial u_{i}}{\partial x_{j}}+\frac{\partial u_{j}}{\partial x_{i}}\right)\right]-\frac{2}{3}\mu \frac{\partial u_{k}}{\partial x_{k}}\delta_{ij},$$ (7)

$$\frac{\partial }{\partial x_{j}}\left(\rho u_{j}C_{p}T-k\frac{\partial T}{\partial x_{j}}\right)=u_{j}\frac{\partial p}{\partial x_{j}}+\left[\mu \left(\frac{\partial u_{i}}{\partial x_{j}}+\frac{\partial u_{j}}{\partial x_{i}}\right)-\frac{2}{3}\mu \frac{\partial u_{k}}{\partial x_{k}}\delta_{ij}\right].$$ (8)

The FVM is a numerical technique that transforms the partial differential equations representing conservation laws over differential volumes into discrete algebraic equations over finite volumes (or elements or cells) (Moukalled et al., 2016). The discretization of the governing Eqs 6–8 in the geometric domain (Figure 1) is carried out using the FVM, based on Ansys Fluent software, with non-overlapping elements as the initial stage in the simulation process. The partial differential equations are then integrated across each discrete element, discretizing or transforming them into algebraic equations that can be solved by an iterative solution. The values of the dependent variables are then determined for each of the elements by solving the system of algebraic equations. The FVM is the dominant approach in CFD simulations due to its innate conservation feature. The FVM’s ability to be formed in a physical space on unstructured polygonal meshes is its most significant feature. Due to these qualities, the finite volume approach has proven to be a very effective tool for the numerical modeling of a wide range of applications involving fluid flow, heat transfer, and mass transfer.

### Mesh-independent investigation

**FIGURE 3:**
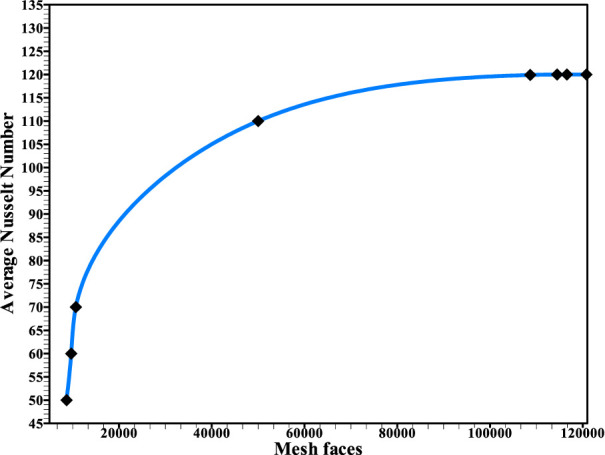
Results of the grid independence test.

The optimum grid face size for the mathematical model was determined *via* a grid-independent analysis. In this study, eight mesh elements are examined with 8,687, 9,687, 10,687, 50,000, 108,687, 114,487, 116,587, and 120,800 cells. On an identical XY graph, the average Nu on the wall of the upper surface of the conduit is depicted using all (eight) mesh faces. As shown in Figure 3 and Table 1, all eight mesh faces produce results close to the average Nusselt number. Therefore, any set of the tested meshes may be used for these eight cases. In this case, the mesh with 114,487 cells is used since it is the best in terms of accuracy and computational time. More cells and a larger mesh may produce better results, but the computation time would be longer as the cell density increases.

**TABLE 1:** Grid independence test for the simulation model.

Mesh faces	Average Nusselt number *N*_*ur*_
8,687	50
9,687	60
10,687	70
50,000	110
108,687	119.9
114,487	119.95
116,587	119.99
120,800	120

### Verification of the numerical code

**FIGURE 4:**
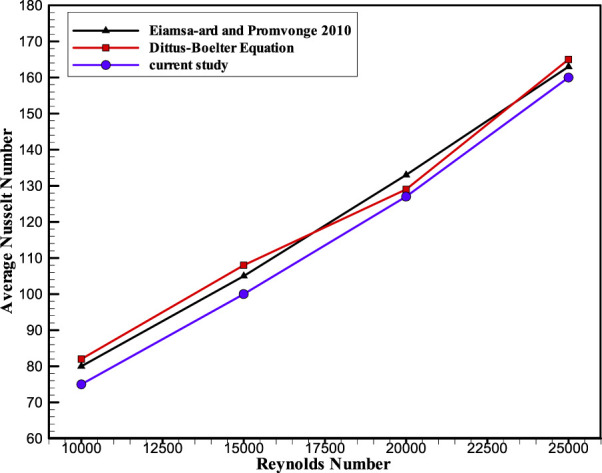
Code validation of the current study.

**FIGURE 5:**
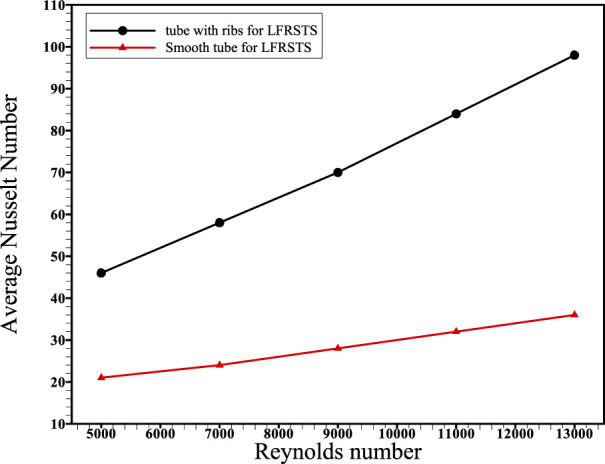
Reynolds numbers and average Nusselt number variation for the absorber tube with transverse ribs and plain absorber tube of the CLFRC system.

To guarantee that the numerical code has been compared to earlier studies and is appropriate for subsequent runs, code validation is required for all numerical activities. This should produce results that are identical to or strikingly similar to those of earlier investigations. Moreover, it is important to understand the capabilities and limitations of any numerical code in addition to its high degree of precision. To validate the computer model, the experimental investigation of thermal properties in round tubes fitted with serrated warped tape conducted by Eiamsa-Ard and Promvonge (2010) was provided. The authors assessed the thermal and flow characteristics of the flow fluid in a round tube to improve heat transmission. Figures 4 and 5 compare simulation solutions for the Nusselt number and Reynolds number to experimental data from Eiamsa-Ard and Promvonge (2010) and the Dittus–Boelter correlation for forced convection inside the conduit. The algebraic outcomes shown in this graph are generally in line with the results of the experimental study by Eiamsa-Ard and Promvonge (2010) and the Dittus–Boelter correlation.

## Results and discussion

The outcomes of CFD simulation present the influence of using transverse ribs inside the absorber tube to enhance the heat transfer and performance of the CLFRC. The mass flow rate and Reynolds numbers were considered to test their impact on the average Nusselt number on the upper absorber tube, friction factor, PEC, and contours of velocity and temperature.

**FIGURE 6:**
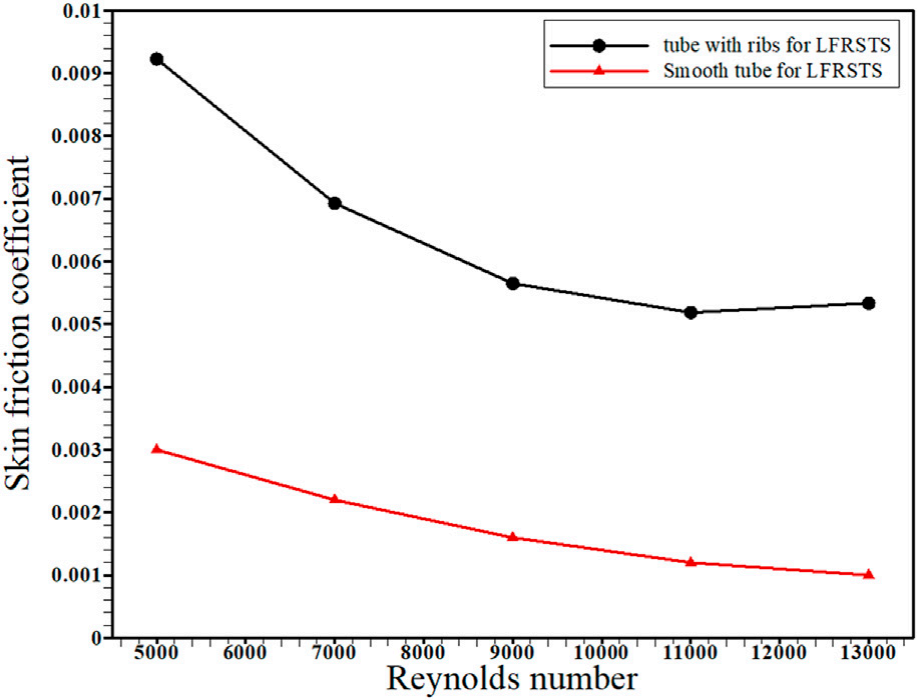
Reynolds numbers and skin friction coefficient variation for the absorber tube with transverse ribs and plain absorber tube of the CLFRC system.

**FIGURE 7:**
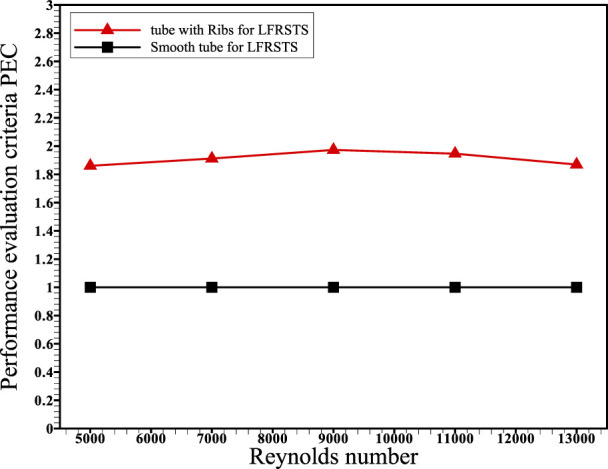
Performance evaluation criterion and Reynolds number variation for the absorber tube with transverse ribs and plain absorber tube in the CLFRC system.

**FIGURE 8:**
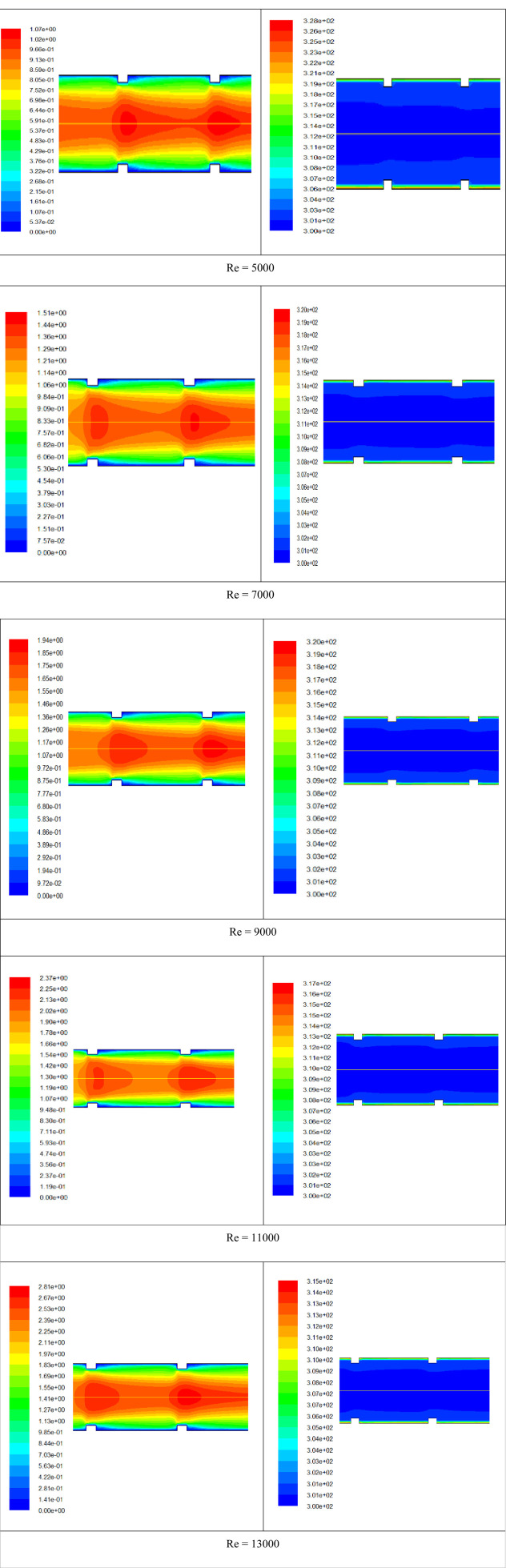
Velocity-contours (m⁄s) (left) and temperature-contours (K) (right) under various Reynolds numbers.

Figure 6 presents the influence of the variation in the Reynolds numbers on average skin friction coefficients for the absorber tube with the transverse ribs and plain absorber tube of the CLFRC system. It is noted that the average skin friction coefficients decrease with increasing Reynolds numbers for both the enhanced absorber tube and the smooth absorber tube. It was detected that the coefficients’ average friction inside the absorber tube with transverse ribs was higher than that of the plain absorber tube in the CLFRC system. The variation in the PEC under different Reynolds numbers using an absorber tube with transverse ribs and a plain absorber tube in the CLFRC system is shown in Figure 7. The results show that the performance evaluation criterion fluctuated between 1.8 and 1.9 with an increase in the Re number. The effects of transverse ribs in the absorber tube in the CLFRC system on the velocity-contours and temperature-contours at various Reynolds numbers are shown in Figure 8. The transverse ribs generate huge mixing and larger vortices close to the ribs, leading to enhancement of the heat transfer coefficients. Maximum mixing fluid and vortices were obtained at the highest Re.

## Conclusion

Transverse ribs in tubes were used to augment the thermal behavior of turbulent flow in the absorber tube with ribs in a CLFRC subject to direct heat flux in the upper pipe. The Nusselt number, friction factor, and PEC were obtained through the CFD model. The following conclusions can be drawn:

1) The average Nusselt number in the absorber tube with transverse ribs in the concentrated linear Fresnel collector is superior to that of the plain absorber tube with different Reynolds numbers.
2) Inclusion of transverse ribs inside the absorber tube improves the average Nusselt number by approximately 115% at Re = 5,000 and 175% at Re = 13,000.
3) The skin friction coefficient for the enhanced absorber tube with ribs in the concentrated linear Fresnel collector is greater than that of the plain absorber tube for all Reynolds numbers.
4) The skin friction coefficient decreases with decreasing Reynolds number, while Nu increases with increasing Reynolds number.
5) The performance evaluation criterion ranges between 1.8 and 1.9 with increasing Reynolds number.

As part of future work, this study could be extended to include the investigation of laminar flow in a linear Fresnel collector by employing different types of nanofluids as heat transfer fluids and different geometries of ribs such as rectangular, trapezoidal, triangular, square, and rectangular.

## Data Availability

The original contributions presented in the study are included in the article/Supplementary Material; further inquiries can be directed to the corresponding authors.
